# Plasmodial HSP70s are functionally adapted to the malaria parasite life cycle

**DOI:** 10.3389/fmolb.2015.00034

**Published:** 2015-06-26

**Authors:** Jude M. Przyborski, Mathias Diehl, Gregory L. Blatch

**Affiliations:** ^1^Parasitology, Philipps University MarburgMarburg, Germany; ^2^Centre for Chronic Disease Prevention and Management, College of Health and Biomedicine, Victoria UniversityMelbourne, VIC, Australia; ^3^Biomedical Biotechnology Research Unit, Department of Biochemistry and Microbiology, Rhodes UniversityGrahamstown, South Africa

**Keywords:** HSP70, HSP40, molecular chaperone, malaria, *Plasmodium falciparum*

## Abstract

The human malaria parasite, *Plasmodium falciparum*, encodes a minimal complement of six heat shock protein 70s (PfHSP70s), some of which are highly expressed and are thought to play an important role in the survival and pathology of the parasite. In addition to canonical features of molecular chaperones, these HSP70s possess properties that reflect functional adaptation to a parasitic life style, including resistance to thermal insult during fever periods and host–parasite interactions. The parasite even exports an HSP70 to the host cell where it is likely to be involved in host cell modification. This review focuses on the features of the PfHSP70s, particularly with respect to their adaptation to the malaria parasite life cycle.

## Introduction

Malaria rates amongst one of the major global health challenges facing developing countries, and over half a million people die from malaria annually, 90% of which are in Africa. *Plasmodium falciparum* causes the most lethal form of human malaria, and nearly all malaria deaths result from infection by this species of *Plasmodium* (World Health Organisation, 2014).

**Figure 1:**
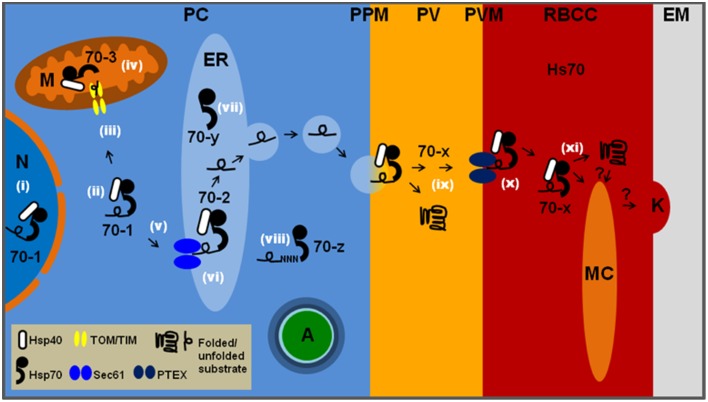
**The location and function of the six PfHSP70s in** ***P. falciparum*****-infected erythrocytes**. PC, parasite cytosol; PPM, parasite plasma membrane; PV, parasitophorous vacuole; PVM, parasitophorous vacuolar membrane; RBCC, red blood cell cytosol; EM, external milieu; N, nucleus; M, mitochondrion; ER, endoplasmic reticulum; MC, Maurer's cleft; K, knob; A, apicoplast; Hs70, human HSP70. All other HSP70s are parasite encoded and labeled as in Table 1. Likely functions at specific cellular localizations are noted by white roman numerals: (i) general proteostasis in the nucleus by 70-1; (ii) general proteostasis in the parasite cytosol by 70-1; (iii) involvement of 70-1 as part of a guidance complex for mitochondrial proteins; (iv) involvement of 70-3 in translocation across mitochondrial membranes followed by protein folding; (v) potential involvement of 70-1 in post-translational ER targeting of secretory proteins; (vi) involvement of 70-2 in protein translocation across PfSec61, followed by general protein quality control via the unfolded protein response; (vii) 70-y is likely to function as a NEF for 70-2; (viii) 70-z is likely to function as a NEF for 70-1 and has been shown to be involved in stabilization of asparagine-rich proteins; (ix) 70-x may be involved in maintaining proteins in an unfolded state prior to passage across PTEX in combination with PfHsp101, and also folding of PV resident proteins; (x) both 70-x and Hs70 may be involved in translocation through PTEX followed by protein folding and; (xi) insertion into the Maurer's clefts, knobs and erythrocyte plasma membrane. Many of the above processes are likely to require co-chaperone activity provided by various PfHSP40s.

HSP70 chaperones and their HSP40 co-chaperones are critical to the maintenance of cellular proteostasis through their role in the folding, refolding, aggregation suppression, translocation, and degradation of proteins. *P. falciparum* heat shock protein 70 (PfHSP70) proteins are proposed to play a major role in parasite development and survival, particularly within the human host (Shonhai et al., 2007, 2011; Njunge et al., 2013; Pesce and Blatch, 2014). Malaria parasites invade human hepatocytes and erythrocytes, and have adapted to their intracellular host environment, particularly to stresses such as the temperature fluctuations associated with febrile episodes. Part of this adaptation has been to evolve an optimal arsenal of six PfHSP70s (Table 1), half the number found in humans and other eukaryotic system, including other parasites (Shonhai et al., 2011). To nevertheless allow interaction with a large array of potential substrates, it appears that the parasite has increased its HSP40 co-chaperone complement to 49 homologs (Botha et al., 2007; Pesce and Blatch, 2014). This suggests that the plasmodial PfHSP70–PfHSP40 chaperone system is an extreme example of HSP70 general chaperone capacity that is highly specified through its HSP40 chaperone partners. The PfHSP70s have been more extensively studied than the PfHSP40s, and although progress has been made in recent years, we still know relatively little about PfHSP70–PfHSP40 partnerships (Pesce and Blatch, 2014). Five of the PfHSP70s are located, or predicted to be located, within the parasite (PfHsp70-1, PfHsp70-2, PfHsp70-3, PfHsp70-y, and PfHsp70-z) and one is secreted to the parasitophorous vacuole (PV) and further exported to the host cell (PfHsp70-x; Table 1; Figure 1). Orthologs of the five parasite-resident PfHSP70s are found in all *Plasmodium* species, and very likely play a fundamental role in proteostasis of the subcellular compartments that they occupy. In contrast, PfHsp70-x is only found in those *Plasmodium* species with highly expanded exportomes (*P. falciparum* and *P. reichenowi*), suggesting a specialized function in protein trafficking or folding (Külzer et al., 2012). This review focuses on the chaperone properties of the six PfHSP70s, particularly with respect to their adaptation to the malaria parasite life cycle.

**Table 1:** **Accession numbers, localization, and properties of** ***P. falciparum*** **HSP70 homologs**.

**Protein**	**PlasmoDB accession**	**Localization**	**Properties**
PfHsp70-1	PF3D7_0818900	Cytosol, nucleus	Protein folding and protein aggregation suppression. C-terminal EEVD sequence for interaction with PfHOP and possibly other partners
PfHsp70-2	PF3D7_0917900	ER	BiP, grp. N-terminal signal sequence and C-terminal ER retrieval sequence. Translocation of proteins into the ER and retrograde translocation of proteins for degradation
PfHsp70-3	PF3D7_1134000	Mitochondria?	Predicted N-terminal mitochondrial transit peptide. Translocation of proteins into the mitochondrion?
PfHsp70-x	PF3D7_0831700	PV, host cell	N-terminal signal sequence and non-PEXEL export sequence. C-terminal EEVN. Associated with J-dots. Trafficking of specialized virulence proteins
PfHsp70-y	PF3D7_1344200	ER?	HSP110, likely NEF for PfHsp70-2. N-terminal signal sequence and C-terminal ER retrieval sequence
PfHsp70-z	PF3D7_0708800	Cytosol?	HSP110, likely NEF for PfHsp70-1. Suppression of the aggregation of asparagine repeat-rich proteins

*BiP, immunoglobulin binding protein; ER, endoplasmic reticulum; grp, glucose regulated protein; Hop, HSP70/HSP90 organizing protein; NEF, nucleotide exchange factor; PEXEL, Plasmodium export element; PV, parasitophorous vacuole*.

## Proteostasis of the cytoplasm and the nucleus: PfHp70-1 and PfHsp70-z

The parasite resident PfHsp70-1 exhibits key features that suggest that it is uniquely adapted to provide cytoprotection under stressful conditions such as febrile episodes. In particular, PfHsp70-1 has a thermostable C-terminal domain that is proposed to stabilize the overall conformation of the protein (Misra and Ramachandran, 2009), making it more thermostable than the human HSP70. Furthermore, it has the properties of a typical molecular chaperone (Matambo et al., 2004; Ramya et al., 2006) and has been shown using *in vitro* assays (Shonhai et al., 2008; Cockburn et al., 2011) and *in vivo* assays in bacteria (Shonhai et al., 2005) and yeast (Bell et al., 2011) to be efficient at suppressing protein aggregation. There are high levels of this molecular chaperone throughout the erythrocytic stages of the parasite life cycle (Acharya et al., 2009), with increased levels at febrile temperatures (Kumar et al., 1991; Joshi et al., 1992; Pesce et al., 2008). The localization of PfHsp70-1 to the parasite nucleus and cytosol (Table 1; Figure 1; Kumar et al., 1991; Pesce et al., 2008) suggests that it is an agent of proteostasis within these subcellular compartments. In addition, PfHsp70-1 could be important in keeping certain proteins in an unfolded translocation-competent state; such as proteins destined for the mitochondria. There are eight cytoplasmic PfHSP40s (Njunge et al., 2013), and there is experimental evidence for an interaction of PfHsp70-1 with Pfj4 (PF3D7_1211400; Pesce et al., 2008), PfHsp40 (PF3D7_1437900; Botha et al., 2011), and PFB0595w (PF3D7_0213100; Njunge et al., 2015). The other cytoplasmic PfHSP40s include proteins predicted to be ribosome-associated, membrane-associated and involved in diphthamide biosynthesis (Njunge et al., 2013). Therefore, a range of different unrelated protein substrates could potentially be delivered to PfHsp70-1 through these cytoplasmic co-chaperones.

PfHsp70-z belongs to the HSP110 family and is likely to act as a nucleotide exchange factor for PfHsp70-1. The protein has recently been shown to be essential for parasite viability, and there is evidence that it is able suppress the aggregation of asparagine repeat-rich proteins more efficiently than its eukaryotic orthologs (Muralidharan et al., 2012). Proteins containing asparagine repeats are prone to aggregation, and the proteome of the malaria parasite is rich in these proteins. PfHsp70-z may have evolved to protect the malaria parasite against the harmful effects of these proteins during febrile episodes, and this is supported by the recent finding that, similarly to PfHsp70-1, PfHsp70-z is also a thermostable molecular chaperone (Zininga et al., 2015). Therefore, PfHsp70-1 and PfHsp70-z together ensure that proteostasis is maintained in the parasite cytoplasm (Table 1; Figure 1).

## Protein secretion and degradation: PfHsp70-2/PfBiP and PfHsp70-y

Proteins bearing a hydrophobic N-terminal signal sequence are routed via the endoplasmic reticulum (ER) to the PV (Adisa et al., 2003), unless they possess further targeting signals (Deponte et al., 2012). The ER-based HSP70, PfHsp70-2 (also called *P. falciparum* immunoglobulin binding protein, PfBiP; and *P. falciparum* glucose regulated protein, Pfgrp; Kumar et al., 1988, 1991; Kumar and Zheng, 1992) has not been extensively studied, but is very likely to be involved in protein secretion and degradation processes associated with the ER (Table 1; Figure 1). Similarly to PfHsp70-1, PfHsp70-2 has been shown to exhibit chaperone properties. Using *in vitro* assays PfHsp70-2 was found to protect alcohol dehydrogenase and glutamate dehydrogenase from thermally-induced unfolding (Ramya et al., 2006). PfHsp70-2 and associated co-chaperones (e.g., the membrane bound PfHSP40, PfSec63/PF3D7_1318800) have been proposed to be involved in the secretory pathway, working closely with the translocon machinery (PfSec61 complex; Tuteja, 2007). Analysis of the components of the PfSec61 complex suggests the existence of a signal recognition particle-based co-translational ER translocation mechanism, similar to the mammalian system (Zimmermann and Blatch, 2009); however, a post-translational mechanism cannot be excluded. ER proteins misfolded beyond repair are probably removed by retrograde translocation, possibly using components of the ER-associated protein degradation pathway (ERAD), which have previously been identified in the parasite (Spork et al., 2009). PfHsp70-2 may work together with an ER-luminal PfHSP40 (e.g., Pfj2/PF3D7_1108700; Botha et al., 2007; Pesce et al., 2010) in such a quality control process, similar to the human BiP-ERdj5 system (Hagiwara and Nagata, 2012). PfHsp70-y, similar to PfHsp70-z, belongs to the HSP110 family, and is thus likely to function as a NEF for PfHsp70-2. Although this protein contains a predicted apicoplast targeting signal, it also contains a C-terminal ER retrieval sequence which seems to be dominant (Heiny et al., 2012). Thus, it appears unlikely that this protein is actually targeted to the apicoplast, but is rathermore likely to be ER localized.

## Import into organelles: PfHsp70-3

*Plasmodium* parasites contain both a mitochondrion and a secondary plastid, referred to as the apicoplast. Protein import to these compartments is post-translational, and thus proteins must be kept in an unfolded state prior to membrane translocation. In other systems, organellar HSP70s provide assistance in this process. Indeed, *P. falciparum* is predicted to encode a mitochondrial HSP70, PfHsp70-3 (Njunge et al., 2013; Table 1; Figure 1). Although not yet experimentally addressed, it is likely that cytosolic PfHsp70-1 also contributes to mitochondrial protein import, possibly as part of a chaperoning guidance complex which keeps proteins in a translocation competent state prior to passage across the mitochondrial membranes. Based on other systems, it is likely that PfHsp90 and Pf14-3-3 are also part of this complex.

Several studies have suggested that PfHSP70s may also play a role in transport of proteins to the apicoplast, a secondary endosymbiotic organelle which is related to chloroplasts (Foth et al., 2003; Misra and Ramachandran, 2009). However, so far no evidence has verified the presence of a HSP70 homolog within the apicoplast. As all other PfHSP70 homologs have been localized to other cellular compartments, it seems unlikely that PfHSP70s play a direct role in translocation across apicoplast membranes. Nevertheless, the observation that inhibitors of HSP70 cause a delay in apicoplast protein transport (Ramya et al., 2006) suggests that possibly PfHsp70-2 (PfBiP) may be involved in chaperoning client proteins prior to passage across the SELMA (symbiont-derived ERAD-like machinery) translocon which is believed to be the gateway to the apicoplast (Hempel et al., 2007; Spork et al., 2009). This interpretation is supported by studies in the related apicomplexan, *Toxoplasma gondii*, which identified TgBiP as an important *trans* factor in protein targeting to the apicoplast (Yung et al., 2003). Although as yet unproven, it has been suggested that apicoplast targeted HSP60 (cpn60; GroEL) or HSP100 (ClpC) may have taken over the function of HSP70 in protein translocation across the multiple apicoplast membranes (Sato, 2011). If this is true, it represents a fascinating evolutionary example of a parasite “losing” one chaperone and co-opting another to take over its function.

## Protein export: PfHsp70-x

*P. falciparum* is predicted to export over 450 proteins (8% of the entire proteome) into the human erythrocyte where they are involved in modifications of the host cell which are essential for parasite survival (Hiller et al., 2004; Marti et al., 2004; Sargeant et al., 2006; Maier et al., 2008). The protein export pathway involves: (i) entry into and transit through the ER; (ii) secretion into the PV; (iii) translocation across the parasitophorous vacuolar membrane (PVM) into the erythrocyte; (iv) trafficking of soluble proteins to their final destination in the cytosol; or (v) trafficking of membrane proteins in complexes or through vesicular networks to the Maurer's Clefts and to the plasma membrane of the erythrocyte (Deponte et al., 2012; Figure 1). Therefore, any exported parasite protein must cross a number of different membranes in an unfolded translocation-competent conformation by a process that will very likely require molecular chaperones at all stages. Two classes of exported proteins have been identified. One class contains a so-called *Plasmodium* export element or host targeting (PEXEL/HT) motif downstream of an N-terminal ER-type hydrophobic signal sequence. The PEXEL/HT motif is cleaved in the ER (Chang et al., 2008; Boddey et al., 2010; Russo et al., 2010) and (in a process not yet understood) directs proteins into an export pathway to the host cell. Another class of proteins, so called PEXEL Negative Exported Proteins (PNEPs) do not contain any recognizable conserved export motif, and indeed often do not contain an N-terminal ER-type signal sequence (Spielmann and Gilberger, 2010; Heiber et al., 2013). A notable member of the PNEPs is *P. falciparum* erythrocyte membrane protein 1 (PfEMP1) which is transported to the host cell plasma membrane, and that is directly implicated in malaria pathology by a process called cytoadherance. Despite divergence in the signals mediating export, the export pathway of PEXEL and PNE proteins appears to converge at a protein translocon within the PVM, referred to as PTEX (*Plasmodium* translocon of exported proteins; de Koning-Ward et al., 2009; Gruring et al., 2012). Thus, down-regulation or functional interruption of a PTEX component leads to a transport block of both PEXEL and PNE proteins (Beck et al., 2014; Elsworth et al., 2014). Additionally, inhibition of protein unfolding also leads to a transport block of both protein classes in the PV (Gehde et al., 2009; Gruring et al., 2012). Recently, PfHsp70-x was shown to be localized to both the PV and host cell cytosol (Table 1; Figure 1). Within the host cell cytosol, PfHsp70-x could be localized to small punctate structures known as J-dots (Külzer et al., 2012). These structures are motile within the host cytosol, and have also previously been shown to contain PfEMP1 (Külzer et al., 2010). Thus, the possibility exists that PfHsp70-x is involved in protein traffic both (a) across the PV, possibly as an associated factor of the PTEX translocon, and (b) within the host cell cytosol, possibly as part of a chaperoning complex to carry proteins to their final sub-cellular localization. A role for PfHsp70-x within the PV would make sense, as the PTEX translocon is partially composed of PfHsp101, a molecular chaperone known (in other systems) to often functionally associate with HSP70s. Alternatively, in the PV PfHsp70-x may be involved in keeping exported proteins unfolded prior to passage across the PVM via PTEX. Once proteins reach the *trans* side of the PVM, they need to be folded and transported to their final destination. PfHsp70-x may be involved in this process, as may residual human HSP70s. Additionally, PfHsp70-x may be required to chaperone larger proteins complexes through the erythrocyte cytosol, and assist in their insertion into either the Maurer's clefts, or host cell plasma membrane. Of note, PfHsp70-x is only encoded by parasite species which export EMP1-like proteins, suggesting that it may play a special role in export of this specialized (but highly important) protein family (Külzer et al., 2012).

## PfHSP40s

Although this review concentrates on PfHsp70s, it would be unwise not to comment on the HSP40s. HSP40s function as co-chaperones for HSP70s and are involved in specificity of substrate recognition and stimulation of HSP70 ATP hydrolysis activity. Of all the parasite-resident PfHSP40s, so far only Pfj4 has been captured in a common complex with PfHsp70-1 using immunoprecipitation assays (Pesce et al., 2008). Interestingly, there is evidence from gel filtration studies that PfHsp70-1 may occur in two distinct complexes, one with Pfj4 and another with PfHsp90 (Pesce et al., 2008). This PfHsp90–PfHsp70-1 complex may include and be functionalized by *P. falciparum* HSP70/HSP90 organizing protein (PfHOP) (Gitau et al., 2012).

Amazingly, in addition to exporting PfHsp70-x, *P. falciparum* may export almost half (23) of its expanded complement of 49 HSP40s (Sargeant et al., 2006; Botha et al., 2007). Although the exact nature of PfHSP70–PfHSP40 pairings has yet to be investigated in detail, localization data suggests that at least two of the exported PfHSP40s colocalize and are found in a complex with PfHsp70-x and thus may by involved in regulating PfHsp70-x function (Külzer et al., 2012). Several other exported HSP40s have been implicated in host cell modification, including biogenesis of knob structures under the surface of the erythrocyte, insertion of PfEMP1 into these structures and alteration of erythrocyte stiffness, all features which are likely to have a direct effect on pathogenicity of parasites in the human host (Maier et al., 2008). It is however not yet known whether these proteins carry out their function mainly in conjunction with PfHsp70-x, or possibly together with residual human HSP70 which is found in high concentrations within the host cell, a fascinating area of research which deserves further study. A recent publication suggests that exported HSP40s may be able to functionally associate with both PfHsp70-x and human HSP70, further increasing the flexibility of the potential chaperone-cochaperone pairing (Hatherley et al., 2014). So far, *in silico* data suggest that *P. falciparum* has evolved to use a minimal complement of PfHSP70s, supported by an expanded HSP40 complement to confer substrate specificity and diversity. Deciphering the role and functional interactions of the expanded PfHSP40 complement will allow us a window into understanding this evolutionary flexibility.

## PfHSP70s as drug targets

Although counter-intuitive due to the high sequence conservation amongst members of the HSP70 family from highly divergent organisms, they may actually be specially suited as drug targets. Such highly conserved proteins are often essential for cell survival, a key factor for any potential drug target. Furthermore, these proteins evolve considerably slower than non-conserved protein families, leading to less variation under drug selection pressure, and hence less chance of the emergence of drug resistance (Edkins and Blatch, 2012). However, there is sufficient structural and functional variation among HSP70s of different origins, for them to be specifically inhibited (Shonhai et al., 2007; Pesce et al., 2010; Shonhai, 2010). Due to interest in their potential as anti-cancer medication, a growing number of HSP70 inhibitors are being made available. Indeed, certain features of HSP70s make them amenable to functional inhibition (and hence druggable): such as their ability to bind small molecules that can be readily detected in simple assays (e.g., ATP); and their association with regulatory factors (e.g., ATP hydrolysis stimulation by HSP40s) whose inhibition has detectable functional consequences. A number of different compound classes have been identified as modulators of HSP70 activity, including ATP mimics, spergualins, pyrimidinones, and peptides (Zininga and Shonhai, 2014). PfHsp70-1 has also been found to be modulated by a number of different classes of small molecules. Pyrimidinones have been shown to significantly inhibit the ATPase activity of PfHsp70-1 as well as the *in vitro* growth of the malaria parasite (Chiang et al., 2009). Further studies on these pyrimidinones have shown that they are able to modulate both the basal and HSP40-stimulated *in vitro* ATPase activities of PfHsp70-1 (Botha et al., 2011). PfHsp70-1 and PfHsp70-x are able to efficiently suppress the aggregation of an aggregation-prone model substrate (malate dehydrogenase) in a thermal denaturation-based assay (Cockburn et al., 2011, 2014). A number of natural product compounds showing anti-plasmodial activity were found to selectively modulate the *in vitro* protein aggregation suppression activity of PfHsp70-1 (1,4-naphthoquinones and marine prenylated alkaloids or malonganenones; Cockburn et al., 2011). Some of these compounds were also found to selectively modulate the *in vitro* protein aggregation suppression activity of PfHsp70-x, as well as the basal and HSP40-stimulated ATPase activities of both PfHsp70-1 and PfHsp70-x, compared with human HSP70 (Cockburn et al., 2014). Therefore, PfHsp70-1 and PfHsp70-x appear to be druggable; but a much more concerted effort in drug discovery is needed before the first drug hits the market.

## Conclusion

Although detailed functional research into the HSP70 chaperone complement of *P. falciparum* is still in its infancy, we have already begun to notice small but important differences between this parasite and other model systems. This knowledge has already been applied to identify small molecules which differentially inhibit PfHSP70s compared to their human counterparts. A deeper knowledge of the mechanistic peculiarities of PfHSP70s, especially with regard to their HSP40 co-chaperones, is likely to reveal further unusual features of this chaperone system which may be amenable to drug-design strategies. To this end, important progress is being made by recombinant expression and purification of all the PfHSP70s and potential PfHSP40s for functional assays. The reduced HSP70 complement of the parasite suggests that certain members have evolved to carry out a larger number of functions than their orthologs in other species. This may represent a “chaperone bottleneck” which can be targeted by selective inhibitor design.

## Author contributions

GB and JP contributed by drafting and critically revising the manuscript. MD contributed by conceptualizing and rendering Figure 1 and critically revising the manuscript.

### Conflict of interest statement

The authors declare that the research was conducted in the absence of any commercial or financial relationships that could be construed as a potential conflict of interest.
